# Two Complete Sequences for Genomes of Human Respiratory Syncytial Virus Genotype ON1 Field Strains from Beijing, China

**DOI:** 10.1128/genomeA.01042-17

**Published:** 2017-10-12

**Authors:** Guanglin Cui, Runan Zhu, Jie Deng, Yuan Qian

**Affiliations:** Laboratory of Virology, Beijing Key Laboratory of Etiology of Viral Diseases in Children, Capital Institute of Pediatrics, Beijing, China

## Abstract

Here, we report the complete sequences for genomes of two human respiratory syncytial virus field strains. It was determined by sequence alignment and phylogenetics that they belong to genotype ON1, characterized by a 72-nucleotide duplication in the C termini of the attachment protein G genes.

## GENOME ANNOUNCEMENT

Human respiratory syncytial virus (hRSV) is the most important viral pathogen causing severe respiratory tract infections, especially in pediatric patients worldwide (1). The genome of hRSV consists of a nonsegmented, single-stranded, negative-sense RNA of about 15 kb and contains 10 genes encoding at least 11 proteins, including nucleocapsid protein (N), matrix protein (M), phosphoprotein (P), attachment glycoprotein (G), fusion protein (F), polymerase protein (L), two viral membrane proteins (M2-1 and M2-2), two nonstructural proteins (NS1 and NS2), and a small hydrophobic protein (SH). HRSV can be divided into two groups (hRSV-A and hRSV-B) by antigenic or genetic analysis.

Genotype ON1, characterized by a 72-nucleotide duplication in the C-terminal part of the G gene, is 1 of 11 hRSV-A genotypes (GA1 to GA7, SAA1, NA1 and NA2, and ON1). It was first identified in nasopharyngeal swabs collected on 29 December 2010 (2). To date, identification of genotype ON1 around the world and rapid replacement of the prevailing genotype by ON1 have been reported (3).

It is reported here that complete sequences from two field strains of genotype ON1, BJ/40180 and BJ/45117, were determined. They were isolated from two children hospitalized with acute respiratory illness at the Affiliated Children’s Hospital, Capital Institute of Pediatrics in Beijing, China. The child infected with BJ/40180 was a 5-month-old boy, while the other child with BJ/45117 was a 17-month-old girl. The procedures for nasopharyngeal aspirate collection, virus isolation, RNA extraction, and reverse transcription were the same as those used in previous studies by this group (3, 4). After determining the presence of the 72-nucleotide duplication in the G gene by a PCR method developed previously (4), 15 contigs covering the whole genome of hRSV were amplified by using 15 pairs of primers described previously (5). Because of failure of amplification of the fifth and the seventh segments, two additional forward primers, 5′-GCTTGGTGGTGGTTTTCTTTCCAGG-3′ and 5′-TAATGTGACTGGTGTGCTTCTGGC-3′, were designed based on the sequencing results of the fourth, the sixth, and the eighth segments. The PCR products were sent to a service company for sequencing (Invitrogen, Beijing, China) using cycle sequencing in the forward and reverse directions. Chromatogram files were inspected using Chromas Lite 2.1 (Thehnelysium, South Brisbane, Australia). Sequence segments were assembled with DNASTAR 5.01 (DNASTAR, WI).

The complete genomes of strains BJ/40180 and BJ/45117 were 15,231 and 15,236 nucleotides, respectively. Analysis by sequence alignment with the prototype Long strain confirmed that there were 72-nucleotide duplications in the G genes from both strains. Phylogenetic analysis indicated that they belonged to genotype ON1. Using the South Korean strain RSVA/GN435/11 (GenBank accession number JX627336) as a mapping reference sequence, 11 open reading frames encoding NS1, NS2, N, P, M, SH, G, F, M2-1, M2-2, and L proteins were annotated. The sequences were submitted by using the NCBI Sequin tool.

### Accession number(s).

The whole-genome sequences of strains BJ/40180 and BJ/45117 have been deposited in GenBank under the accession numbers MF614946 and MF614947, respectively.
